# The Future of Personalized Medicine in Space: From Observations to Countermeasures

**DOI:** 10.3389/fbioe.2021.739747

**Published:** 2021-12-13

**Authors:** Elizabeth Pavez Loriè, Sarah Baatout, Alexander Choukér, Judith-Irina Buchheim, Bjorn Baselet, Cinzia Dello Russo, Virginia Wotring, Monica Monici, Lucia Morbidelli, Dimitri Gagliardi, Julia Caroline Stingl, Leonardo Surdo, Vincent Lai Ming Yip

**Affiliations:** ^1^ Leibniz Institute for Environmental Medicine, IUF, Düsseldorf, Germany; ^2^ Radiobiology Unit, Belgian Nuclear Research Centre (SCK CEN), Mol, Belgium; ^3^ Department of Biotechnology, Ghent University, Ghent, Belgium; ^4^ Laboratory of Translational Research “Stress and Immunity”, Department of Anesthesiology, Hospital of the Ludwig-Maximilians-University, Munich, Germany; ^5^ Department of Healthcare Surveillance and Bioethics, Section of Pharmacology, Università Cattolica Del Sacro Cuore, Fondazione Policlinico Universitario A. Gemelli IRCCS, Rome, Italy; ^6^ MRC Centre for Drug Safety Science and Wolfson Centre for Personalized Medicine, Institute of Systems, Molecular and Integrative Biology (ISMIB), University of Liverpool, Liverpool, United Kingdom; ^7^ International Space University, Illkirch-Graffenstaden, France; ^8^ ASA Campus Joint Laboratory, ASA Research Division, Department of Experimental and Clinical Biomedical Sciences, University of Florence, Florence, Italy; ^9^ Department Life Sciences, University of Siena, Siena, Italy; ^10^ Manchester Institute of Innovation Research, Alliance Manchester Business School, The University of Manchester, Manchester, United Kingdom; ^11^ Institute of Clinical Pharmacology, University Hospital of the RWTH Aachen, Aachen, Germany; ^12^ Space Applications Services NV/SA for the European Space Agency, Noordwijk, Netherlands

**Keywords:** personalized medicine, space biology, *in vitro* modelling, immunology, radiation, dermatology, future approach, pharmacology

## Abstract

The aim of personalized medicine is to detach from a “one-size fits all approach” and improve patient health by individualization to achieve the best outcomes in disease prevention, diagnosis and treatment. Technological advances in sequencing, improved knowledge of omics, integration with bioinformatics and new *in vitro* testing formats, have enabled personalized medicine to become a reality. Individual variation in response to environmental factors can affect susceptibility to disease and response to treatments. Space travel exposes humans to environmental stressors that lead to physiological adaptations, from altered cell behavior to abnormal tissue responses, including immune system impairment. In the context of human space flight research, human health studies have shown a significant inter-individual variability in response to space analogue conditions. A substantial degree of variability has been noticed in response to medications (from both an efficacy and toxicity perspective) as well as in susceptibility to damage from radiation exposure and in physiological changes such as loss of bone mineral density and muscle mass in response to deconditioning. At present, personalized medicine for astronauts is limited. With the advent of longer duration missions beyond low Earth orbit, it is imperative that space agencies adopt a personalized strategy for each astronaut, starting from pre-emptive personalized pre-clinical approaches through to individualized countermeasures to minimize harmful physiological changes and find targeted treatment for disease. Advances in space medicine can also be translated to terrestrial applications, and vice versa. This review places the astronaut at the center of personalized medicine, will appraise existing evidence and future preclinical tools as well as clinical, ethical and legal considerations for future space travel.

## Introduction

Space flight exposes humans to extreme physical and environmental conditions. The environmental challenges include acceleration forces, confinement, isolation, microgravity and radiation exposure. Initial effects on the human body are space motion sickness, headaches, congestion and lower back pain as a result of adaptation to microgravity. Ill health can limit the mission performance of space crews, cosmonauts, taikonauts or astronauts (the term “astronaut” will be used representatively in this article). Longer duration missions are associated with immune dysregulation, radiation-induced changes, cardiovascular and muscle deconditioning as well as bone loss (Stepanek et al., 2019). As a consequence of these physical challenges, the medical standards for astronaut selection are rigorous. A review of United States (US) astronaut selection between 1981 and 2011 identified 26% of finalist applicants who were rejected on medical grounds. The most common causes for medical disqualification were disorders in the following categories: visual (38%), cardiovascular (14%) and psychiatric and behavioral (9%) (Johnston et al., 2014).

“Space medicine” is a broad clinical discipline responsible for astronauts’ health. This includes pre-mission screening to prevent disease, health care delivery during missions and long-term recovery and restoration of health post-mission (Hodkinson et al., 2017). Countermeasures aim to protect the health of astronauts from the harmful effects of space flight. Examples include mitigation of radiation exposure by scheduling missions during low solar activity periods and potentially medicines as radiation protectants (McLaughlin et al., 2017). Resistive exercise is recommended to maintain bone health through stimulation of osteogenesis (Guadalupe-Grau et al., 2009). Despite comprehensive medical standards astronauts can still experience injury, ill health and medical emergencies during space flight. The risk of a serious medical event during a mission has been estimated to be around 0.06 per person-year of flight which corresponds to one event every 2.8 years for a crew of six (Komorowski et al., 2016). The risk of injuries coupled with equipment failure increases with the duration of flight and the distance from Earth. On board the International Space Station (ISS), the crew can benefit from a large variety of medical equipment, a regular resupply of medication as well as a direct communication and consultation with the flight surgeon and ground crew. The possibility of health emergency increases due to prolonged exposure to harsher space environmental factors, such as higher radiation energies and doses, which are detrimental not only for the crew but also to the on-board equipment, including medical assets and medications. Communication delay can further reduce the efficiency of the intervention response from ground medical control, thus further increasing health risks for the crew.

Medication use by astronauts has historically been poorly recorded but recent estimates from the ISS suggest that each crewmember has four medications per week (Blue et al., 2019). Response to medications can demonstrate significant variability in terms of efficacy and toxicity for each patient. Traditionally, if a medicine is not effective or resulted in an adverse event an alternative would be prescribed. However, this trial-and-error approach is time-consuming and adverse events may be serious. Around one third of terrestrial medication administrations does not demonstrate the intended efficacy (Spear et al., 2001). Space flight is inherently high-risk, which means treatment failure or adverse events should be avoided as much as possible. Pharmacogenetic screening for astronauts has been proposed as an aspect of personalized medicine that can help to maintain astronaut health (Stingl et al., 2015). As implemented in terrestrial conditions, preemptive pharmacogenetic testing may be an important tool to improve efficacy of a given medication and to avoid side effects (van der Wouden et al., 2017).

As our knowledge of genetics, epigenetics and proteomics has improved the concept of personalized medicine has taken on a more prominent role in research and clinical practice. (Vogenberg et al., 2010a; Vogenberg et al., 2010b). Other components in the development of individualized medicine approaches are the presence of reliable and functional *in vitro* and *in vivo* diagnostic tools which can be used for the optimal selection of treatment solutions to improve the outcome. Here the concept of environmental relevant testing as well as questions with regards to pharmacokinetics (PK) and pharmacodynamics (PD) are very important (Sadee and Dai 2005). The advancements in bioengineering/gene editing, digitalization and big data processing, new precision medicine *in vitro* approaches have started to develop and are gaining ground. One of these is “cellular avatars”, a format that integrates the latest knowledge of induced pluripotent stem cells (iPSCs) and clustered regularly interspaced short palindromic repeats (CRISPR) where cells from individuals are altered to make new cell types or medical conditions which include the person’s “predisposition” (Goetz and Schork 2018). It leads to a lab-on-a-chip based technique where organoids can be developed (van den Berg et al., 2019). Four years ago, a precision cancer care platform was presented, combining whole genome sequencing with a living biobank (in the form of organoids of patients) enabling effective drug screening. This allows the anticipation and prevention of adverse effects or lack of efficacy (Pauli et al., 2017).

**FIGURE 1:**
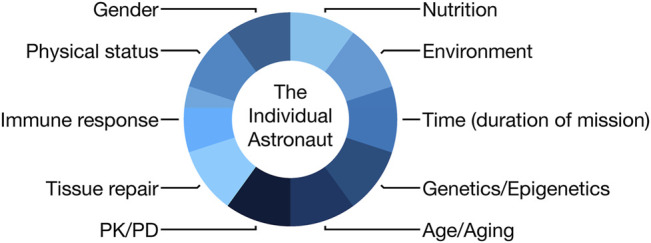
Scheme demonstrating the components of an astronaut that would need to be considered in an individualized medicine approach (PK, pharmacokinetics; PD, pharmacodynamics).

Personalized medicine approaches should be applied to astronauts in order to prevent and minimize harm from space flight, but also to ensure effective diagnosis and treatment of emergent medical problems during missions. Factors to be considered in such a multidimensional practice approach are highlighted in Figure 1. In this review, we will focus on examples and evidence of human space flight-associated health risks from dermatological and immunological observations to radiation exposure detriments, including the use of *in vitro* techniques to facilitate better medical predictions. We will continue by elaborating on the pharmacological aspects of space flight and end by discussing the challenges for personalized medicine in space and potential synergies with terrestrial medicine.

## Examples From Skin, Radiation and Immunology Research in Space

### The Human Skin, an Intricate and Variable First Line of Defense

#### Overview

The human skin connects the outer and inner environments and acts as a barrier protecting the internal organs from the external stressors. It consists of an intricate tissue communication (epidermis, dermis and subcutis) which involves direct connections to the immune, circulatory and nervous system. It functions as: 1) a regulator of water diffusion and temperature, 2) part of immune surveillance, 3) radiation protection, 4) mechanical protection, 5) chemical protection, 6) host to the biosynthesis of vitamins and to receptors for other hormones (Boer et al., 2016). The water retention properties, which function as a natural moisturizer, are partly due to the lipid content of this tissue (Holden et al., 2002). The intercellular lipid space is also key in the diffusion of substances through the skin, playing an important role in drug delivery (Matsui and Amagai 2015). Therefore, the role of a functional epidermis is pivotal for many of the protective and regulating characteristics of the skin. It also contains antimicrobial peptides (AMPs) and other substances that are produced by epidermis residing cells (including immune cells) and by the skin’s own microflora providing a direct defense against pathogens and the upkeep and activation of immune cells (Clausen and Agner 2016; Nakatsuji et al., 2017).

The skin varies in composition and response depending on anatomic region, sex, age, microflora and genetic predisposition resulting in a variation in response to environmental triggers between individuals (Grice et al., 2009; Machado et al., 2010). Of particular interest is the variation in barrier function, regeneration and repair of tissue, especially in extreme conditions such as space flight, where the changes in radiation, gravity levels and spacecraft nanoparticle content can result in skin changes, which can be more easily reverted with “shorter” space missions, but might lead to more permanent changes as the mission duration increases (Guo and Dipietro 2010; Machado et al., 2010). Learning to predict individual astronaut responses to specific environmental triggers and understanding when an exposure transitions from acute to chronic and the biological adaptation becomes the “new” normal, will be important tasks for deep space human flight missions in the future.

#### The Challenges of Space Environment for the Skin

In the terrestrial environment the skin is exposed to biological and chemical stimuli such as weather conditions, ionizing radiation (IR) from the Sun, gravity, pathogens/microbiome and processes such as vitamin D synthesis. These stimuli are absent or significantly modified outside of Earth’s orbit. The ISS is a closed environment which has been accumulating parts of different microflora and particles (antigens) from different crews as well as animals (e.g., mice) over time (Crucian et al., 2016a; Crucian et al., 2016b). Astronauts have to adapt to a new habitat whilst visiting the ISS with reduced hygiene practices. This challenge has been manifested predominantly on the skin as rash and dermatitis. Other skin symptoms reported include dryness, redness, tissue oedema and acne (Crucian et al., 2016b; Dunn et al., 2018; Braun et al., 2019b). The skin also appears to “age” and it is believed to be connected to alterations in the composition of the dermis, which Braun et al. suggested could be linked to anemia and a reduction in oxygen saturation. (Braun et al., 2019b). Skin symptoms could be also linked to dehydration, partly due to insufficient water intake (Lane and Young 2012).

Even though these responses to the ISS environment might be considered as “mild”, they represent underlying biological conditions that can cause accumulation of stress or damage leading to more severe pathology later. Dysregulation of the immune response may be responsible for events such as erythema and dermatitis, but changes in oxygen levels leading to dermal extracellular matrix (ECM) stress are likely to contribute. In one study there was a 15% thinning of the epidermis in three subjects which may have a significant impact on its protective characteristics against environmental stressors (König 2012).

Wound healing presents a challenge in space as it is a vital and intricate process that is dependent on many cells working together and is classically divided into three phases: inflammation, proliferation, and remodeling. These phases have been reported to be altered in weightlessness and unloading (Delp 2008; Monica et al., 2011; Cialdai et al., 2017). Studies from the European Space Agency (ESA) driven expert working group “Tissue healing in space: techniques for promoting and monitoring tissue repair and regeneration” and the projects: “Wound Healing in space: problems and perspectives for tissue regeneration and engineering-WHISPER” and “Wound healing and sutures in unloading conditions-SUTURE IN SPACE”, which focused on surgical wounds, have the goal of providing an insight into tissue repair mechanisms in space flights. The research conducted so far demonstrated that microgravity causes a delay in healing and tissue structure alterations, as well as impairment in fibroblasts migration in wound repair and that platelet rich plasma (PRP) could be used to prevent these changes (Cialdai et al., 2020). PRP is widely used in wound repair and consists of a mixture of growth factors and cytokines obtained from total blood, activating the fraction enriched in platelets (Etulain 2018). This treatment could be used in future space flight, especially longer missions, where medically trained personnel and access to hospital care will be limited.

With regards to the skin microbiome it was reported by Voorhies et al. that there is a shift in the microbial composition seen in all crew members during space flight (Voorhies et al., 2019). There was a significant reduction of Proteobacteria, with an increase in Staphylococcal and Streptococcal species. The decrease in Proteobacteria has also been seen in individuals with atopy (Ruokolainen et al., 2015), which could partially explain the high frequency of skin hypersensitivity reactions/rashes and infections experienced by astronauts (Crucian et al., 2016a).

Skin cancer is considered by many to be like a “wound that won’t heal” (because of the deregulation of VEGF and fibrin deposits in early wound healing) (Dvorak 1986; Dvorak, 2015) and a disease of aging (in which inheritance, damage accumulation and changes in dermal tissue play a role) (Cho 2017). There are no reports of skin cancer developing during missions but the main concerns are related to the long-term effects of radiation on astronaut skin. A study of 312 astronauts from the US showed that there was an increase in non-melanoma skin cancer prevalence suggesting that radiation from space flight could be an important factor for development of skin cancer (Burgdorf and Hoenig 2015). However, it is also important to take into account the amount of underlying and accumulated damage and ultraviolet (UV) exposure before and after the space flights (Chancellor et al., 2014).

Studies that have investigated the effects of microgravity on cancer cells have demonstrated altered lymphocyte response and activation of known oncogenic pathways (e.g., KRAS) (Sahebi and Halvaei, 2017). Future research for skin cancer should focus on factors in human space flight that have the potential to activate and maintain oncogenic pathways. Signs of pre-malignancy in astronaut skin (e.g., Bowen’s disease) and assessment of progression are of importance as together it would indicate if the extra-terrestrial environment (like microgravity, radiation, nanoparticles) together with epigenetic and genetic factors lead to higher cancer risk from space flight.

##### Individual Variation in Skin Response to Space Travel

There are three studies that provide evidence of individual variation in skin response to the space environment. Tronnier et al. demonstrated delayed epidermal proliferation, decreased hydration and increased elasticity and transepidermal water loss (TEWL, capacity the epidermal barrier) during space flight in one test subject (Tronnier et al., 2008). As part of the “Skin B” initiative Braun et al. devised a symptom/survey based report and one based on skin physiological measurements of the same astronauts (*n* = 6) (Braun et al., 2019b; Braun et al., 2019a). In the survey-based study the astronauts reported similar skin symptoms as seen in Tronnier et al. (2008), with the addition of redness and itchiness. In the skin physiology study TEWL, skin hydration and skin thickness were quantitatively evaluated among the six subjects with high variability and contradictory results to the earlier study by Tronnier et al. (2008). Changes to in-flight routines for hygiene and nutrition were unable to explain the interindividual variability alone. These studies highlight the importance of undertaking studies that examine changes at an individual level as well as that of the whole group.

A study of astronaut microbiomes during long duration missions reported diversity between astronaut skin microbiomes (Voorhies et al., 2019). This confirms the link to individual skin-specific properties, which would lead to variations in skin adaptation to different stimuli due to the importance of immune surveillance and barrier function by the skin microbiome. In addition to these observations a recent study reported the prolonged (1 year) skin related problems of one astronaut following mission completion, further highlighting the diversity in skin response (Law et al., 2020).

#### Approaches to Investigate and Develop Treatment Strategies for Human Skin

##### Modelling the Human Skin to Use in Personalized Pre-clinical Approaches

**FIGURE 2:**
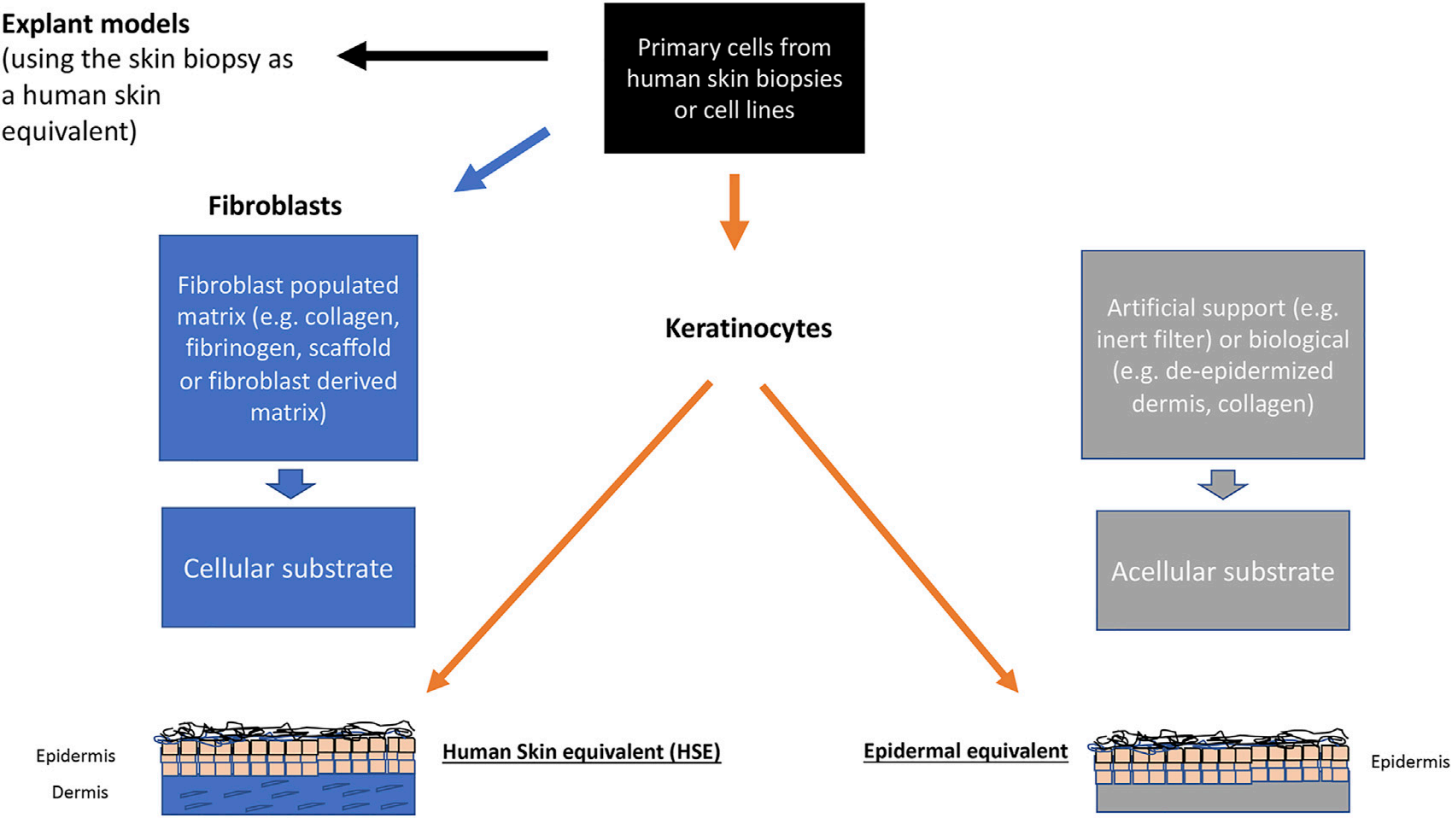
Scheme showing the different *in vitro* modelling techniques in dermatology and skin biology.

At present there are two types of models, the “simple” epidermal models and the more “advanced” two-compartment (two-tissue) skin equivalents (Figure 2). The epidermal models consist mainly of keratinocytes which have been allowed to stratify and keratinize. These models can easily be used in toxicology tests and provide an initial evaluation of the substance impact. This type of model can also include other epidermal homing cells, like melanocytes or immune cells.

The two-compartment models consist of a stromal part, the dermal equivalent (DE), and the epidermis, making a human skin equivalent (HSE). Starting off with de-epidermized dermis or hydrogels (mostly rat tail collagen) with integrated fibroblasts as the basis for the dermal compartment more than 30 years ago, these HSEs largely mimic key elements of human skin biology. Together with further challenging cellular applications such as genetic engineering and cell reprogramming, these HSEs are now indispensable for many applications from basic cell biology to cosmetics, skin aging, up to modelling diseases, i.e., for the fields of physiology, pathophysiology, and regenerative medicine (Ali et al., 2015). Unfortunately, these first-generation HSEs proved to be rather short-lived (three to 4 weeks), which led to new modelling techniques which could provide structural stabilization for longer periods of time. One approach was to replace the rat tail collagen by using fibrin-fibroblast mixture and a non-woven scaffold instead (Stark et al., 2004; Stark et al., 2006). With these dermal equivalents, maintenance of a well stratified and differentiated regenerating epidermis was obtained for several months. Another approach is based on prompted non-collagen, scaffold-free technique that would similarly allow for a DE with an authentic dermal matrix (fibroblast-derived matrix dermal equivalent, fdmDE). Based on work by Ahlfors (Ahlfors and Billiar 2007) a fibroblast-derived matrix was developed serving as a scaffold-free DE (El Ghalbzouri et al., 2009), where the technique of tissue self-assembly was used. These models stand out for an authentic dermal matrix of high mechanical stability and establish a long-lived epidermis which is almost indistinguishable from normal human epidermis *in situ* (Berning et al., 2015; Piredda et al., 2015). In addition to long-term (several months) epidermal regeneration of the skin keratinocytes, this model also proved to be adequate for authentic tumor cell invasion studies by including skin cancer cells (Berning et al., 2015; Piredda et al., 2015).

As presented here there are at least three skin equivalent alternatives (fibrinogen scaffold-based, explant models and fdmDE-based models) in addition to bioprinting techniques (using human based synthetic scaffolds or biodegradable materials) and organ-on- a chip concepts that can be developed to be used in personalized medicine and toxicological testing and should be considered for this task. Nevertheless, it is important to keep in mind the time effectiveness and budget for these models to be used in individualized pre-clinical trials.

Over the years, significant progress has also been made in the preservation and culture of skin explants (*ex vivo* skin models). Using suitable culture conditions, the viability of these explants, which previously declined significantly after about 2 weeks, currently allows their use at temperatures between +4°C and +32°C for over 4 weeks (Hautier et al., 2008; Monici et al., 2018). These *ex vivo* models can be also collected and cryopreserved for over 6 months before use (Tognetti et al., 2017). Furthermore, glycerol preservation can supply decellularized scaffolds (the cells are no longer viable), even if the mechanical properties of the matrix undergo some changes (Wood et al., 2014; Tognetti et al., 2017). These *ex vivo* models offer the advantage of being real human tissues and therefore being able to better represent the complexity of human tissues when the response to various stressors is tested. However, cell viability of explants is dependent on collection/preservation protocols as well as donor conditions such as age and sex (Pianigiani et al., 2016).

##### Approaches to Mimic the Human Skin and Bioengineering in Space and Their Use in Countermeasures

Many studies have investigated the effects of the space environment using human *in vitro* modelling techniques. Some have studied the behavior of immune cells, human fibroblasts, endothelial cells and epithelial cells in simulated micro-gravity or on the ISS (Desai et al., 2005; Morbidelli et al., 2005; Monice et al., 2011; Pietsch et al., 2011; Lu et al., 2017; Cialdai et al., 2020). A review of the literature confirms only two studies performed using skin equivalents, based on the conventional collagen modelling technique (MaTeK). These studies, which were performed on Earth, have given a first insight into changes in tissue proliferation and differentiation (von Neubeck et al., 2015; von Neubeck et al., 2013). As these studies are few and have been performed in a tissue hyperplastic state, without reaching skin homeostasis, it is difficult to draw conclusions other than encouraging more human *in vitro/ex vivo* modelling initiatives. Carrying out experiments of this type is among the activities planned in the SUTURE in SPACE and WHISPER projects supported by ESA.

To include stability and biological accuracy in the equation of modelling, more sophisticated skin equivalents will need to be used. Here the fdmDE technique described above could be an interesting alternative. It would provide space research with a tool to understand important features of skin biology in space. As it is viable for longer periods of time, it can be used to perform longer exposure studies and the fact that it only contains *in situ* produced ECM and tissue structures, makes it a useful substitute to *in vivo* or *ex vivo* human skin. As a reconstitution technique, it also allows us to explore the interaction between different cell types in human skin, by adding or excluding cells from the model. The explant model is another technique that could be used to investigate wound healing (Monici et al., 2018). This old technique is short lived compared to the fdmDE-based skin equivalent, fibroblast derived matrix skin equivalents (fdmSE), but it does provide the opportunity to examine an individual’s skin (with all its components) and may resemble the physical and biomolecular mechanisms of wound repair and inflammation (Riwaldt et al., 2017).

Although significant advances have been made in storage, preservation and culture of skin explants, further efforts should focus on extending the survival of these tissues, thus improving their utilization. In fact, even if the explant technique is the most dated, autologous graft (or allografts in patients lacking skin donor sites, such as those with burns on most of the body), are still considered the gold standard treatment for extensive deep burns and hard-to-heal wounds (Gaucher and Jarraya 2014). An interesting technique is the use of micrografts. They may be obtained autologously, homologously and minimally invasively and have proved to be efficient in promoting tissue regeneration (Trovato et al., 2015; Ceccarelli et al., 2017).

Currently, ESA has projects to develop a 3D bioprinter for the ISS that will be used for generation of cell constructs in microgravity to provide samples for research into e.g. tissue engineering and regenerative medicine. This also opens up the opportunity to model specific events or structures in microgravity. Even though the skin, especially the epidermis, easily assembles itself; bioprinting might be able to reconstruct appendages like hair follicles and sweat glands in microgravity more easily.

The advantage of developing individualized pre-clinical models for space exploration is that the test group is based on a small number of individuals and can be more easily budgeted and developed. The cells can also be transported to the ISS and used for bio grafting (using 3D printing techniques) to improve ther healing of burns or wounds. The scope would be to have models ready to undertake PK and PD testing based on the astronaut’s own skin, either in the form of explant models or with fdmSEs. Due to the expansion of cells from the skin biopsies and their long-term storage, models like the above-mentioned fdmSE can be used and reproduced several times without further burden to the individual. Depending on the research question the models can be adapted to include or exclude different cells. These models would also allow for chronic exposure analysis, paving the way to develop space-specific and individualized medical approaches for each individual and predict biological outcomes of future longer stays outside Earth.

Explant models already show an individualized approach to research, because the technique itself is to cultivate full thickness skin directly without further manipulation. It therefore presents a good alternative to more complex systems. The disadvantage of this technique though is the constant need of new fresh skin for studies and its shorter life-span compared to the fdmSEs, but it is a technique that can be used immediately, whilst waiting for reconstruction models to be established and ready to use.

In addition to tissue or organ specific reconstruction using cells we also need to consider plasma-based nutrients and molecular signaling of these models. Currently, most models either use fetal bovine serum or a plant-based substitute to achieve a good differentiation and maturity of the tissue and organ. But for precision medical approaches and individualized biological research it would be wise to consider the use of human plasma, matching each individual model with its individual nutrient and biochemical components. This would also provide the opportunity to study plasma specific changes.

Moreover, physical factors are important in regulating tissue homeostasis and maturation of tissue constructs. Therefore, both on Earth and in space, it will be necessary to develop advanced bioreactors to better simulate physiological conditions in terms of both biochemical and physical factors.

### Space as an Environment: Radiation

#### Space Radiation: An Overview

**FIGURE 3:**
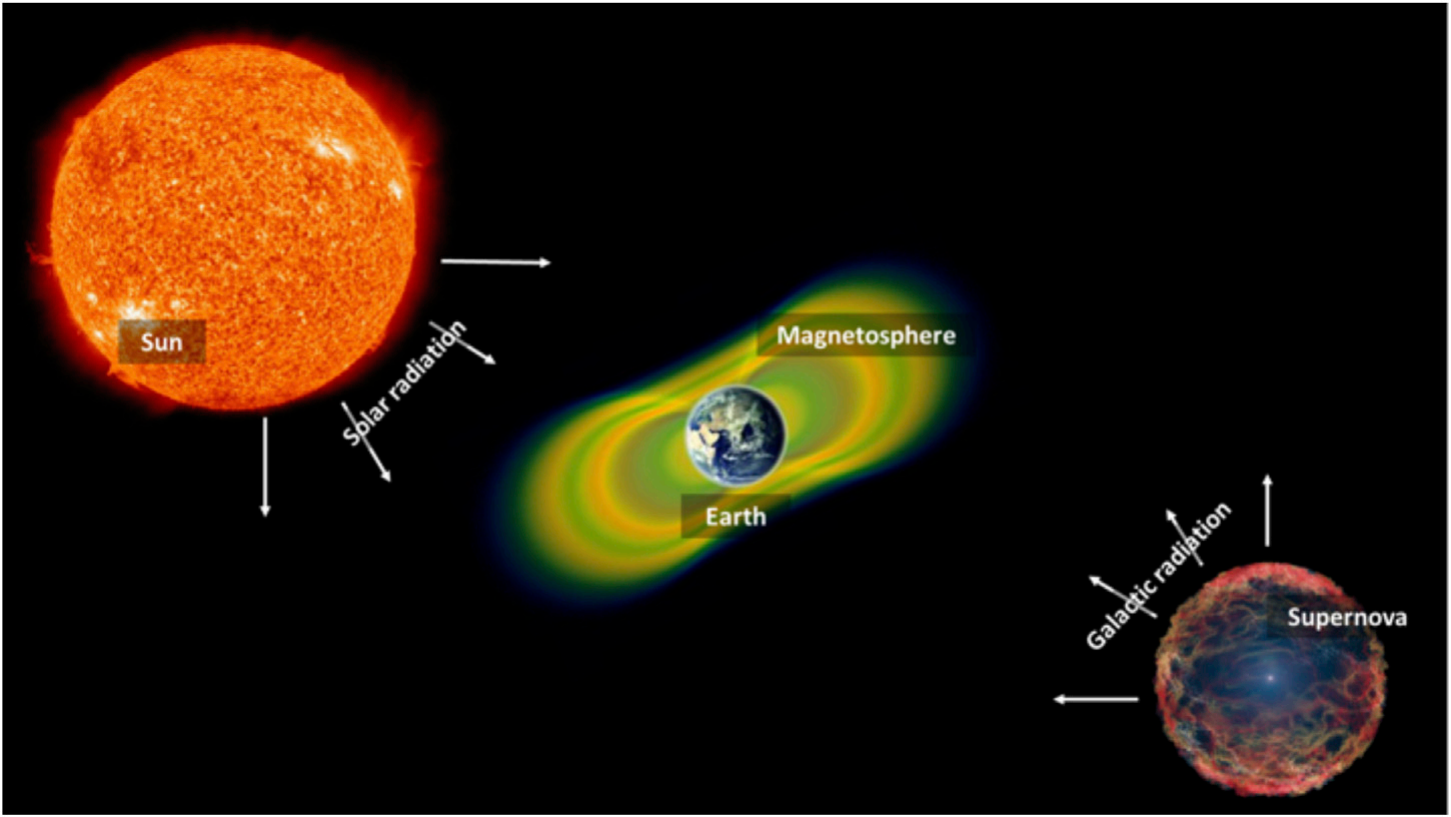
Origins of space radiation. Radiation in space is derived from different sources, such as solar particle events, radiation trapped in our Earth’s magnetosphere as well as radiation coming from distant cosmic events (galactic radiation or galactic cosmic rays).

During their permanence in space, astronauts are exposed to IR. Space radiation differs from the types of radiation experienced on Earth. It consists of atoms in which electrons have been stripped away as they accelerate in interstellar space to velocities close to the speed of light - and in the end, only the nucleus of the atom remains. Space radiation consists of three types: 1) particles that are trapped in the Earth’s magnetic field, 2) particles released into space during solar flares (solar particle events); and 3) galactic cosmic rays, which are composed of high-energy protons and heavy ions originating from outside our Solar System (Figure 3).

In principle, IR interacts along charged particle tracks with biological molecules such as DNA. The process is largely stochastic, and can damage DNA via direct interactions (e.g., ionization and excitation) or via indirect interactions such as through the production of reactive oxygen species (ROS) as a result of radiolysis of water molecules.

There are three main factors that determine the amount of radiation that astronauts receive or how the IR affects astronauts: 1) altitude above ground: at higher altitudes, the protection of the Earth’s atmosphere no longer exists and the magnetic field is weaker, so there is less protection against ionizing particles, and the spacecraft pass through the trapped radiation belts more often. 2) solar cycle: the Sun has a cycle of 11 years, which culminates in a significant increase in the number and intensity of solar flares, especially during periods of many sunspots. 3) Individual susceptibility: genetic as well as epigenetic factors determine what makes one individual more vulnerable to the effects of space radiation than another. The use of biomarkers for radiation sensitivity will be further reviewed here.

#### The Human, Space and Individual Radiation Sensitivity

IR is a well-known cause of negative health effects in humans and the development of preventive measures and guidelines, requires an understanding of the risks of radiation exposure.

Immediate effects of IR exposure are mainly seen in organs with rapidly dividing cells, like the hematopoietic system and linked immune system, gastrointestinal tract, and the skin. However, it also affects the eyes and the reproductive system (Dalci et al., 2004).

With regards to the skin, acute exposure to IR, primarily involves cellular alterations and inflammation. The effects can be seen as erythema, oedema, pigment changes, and depilation. Severe radiation injury results in the complete loss of the epidermis after which the re-epithelialization process begins within 10–14 days after radiation exposure in the absence of infection (McQuestion 2011). It has been shown that space IR also produces a unique cross talk between epidermis and dermis, where a cascade of cytokines and chemokines are secreted in response to these stress activation signals. Here, keratinocytes, fibroblasts, and endothelial cells have been shown to stimulate resident (i.e., LC, DC, mast cells, T cells) and circulating immune cells involving the immune response (Muller and Meineke 2007; Muller and Meineke, 2011).

**FIGURE 4:**
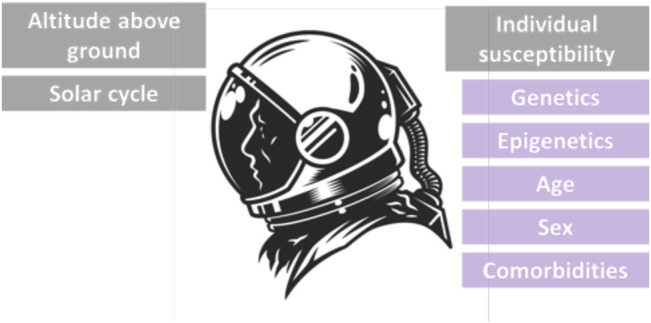
The environmental, physical and individual factors affecting radiation susceptibility of astronauts.

Age, sex, genetic susceptibility, comorbidities, and a variety of other factors (like genetic syndromes, inflammatory state or viral infections) may have an impact on the radiosensitivity of distinct subpopulations too, making the acceptable levels of radiation quite individualized. There is growing evidence of an association between radiation sensitivity and age-related health impacts, including cancer development. Individuals exposed to IR are the most radiosensitive at a young age, which decreases up to maturity, and then increases again at older ages with a higher level of cancer risk. Furthermore, the long-term response to radiation exposure may be influenced by sex-related variables. According to the available evidence, women’s long-term radio-sensitivity is higher than men’s when they are exposed to the same amount of radiation (Figure 4) (ICRP 2007).

Because of advances in our understanding of the space radiation environments inside the spacecraft, on IR effects on tissues, the emergence of new epidemiological data, and changes in the ages and sex makeup of astronauts, NASA (National Aeronautics and Space Administration) has adopted numerous distinct guidelines on radiation limitations since the Apollo era. The high-risk nature of space missions means that radiation protection in manned space flight differs philosophically from that of terrestrial workers. There are guidelines for occupational doses, which have been proposed for NASA to employ in long-term mission design and manned operations. The following is a summary of the approaches that could be used in determining acceptable levels of radiation risk and their possibilities of being effective tools for human long-term deep space missions (Cucinotta 2010).

1) Comparison with occupational fatalities in riskier industries. In riskier industries the number of lives lost as a result of attributable radiation cancer is known to be lower than the number of lives lost as a result of most other occupational deaths. Furthermore, because of continuing advancements in ground-based occupational safety during the last 20 years, this comparison would be quite restricted for ISS operations or Lunar and Mars missions at this time and might not give a broad comparative setup.
2) Analysis of cancer rates in the general population. Radiation-induced cancer can result in a significant loss of life compared to cancer fatalities in the general population, which often occur after the age of 70. Therefore, the astronaut population would be compared to the general population to detect radiation risk levels.
3) Doubling dose for a period of 20 years after exposure. During the worker’s career, this provides a roughly equal reference foundation of life-loss from other occupational risks or cancer mortality background. However, it ignores the importance of mortality later in life.
4) Using a 3% ground-based work limit or a comparable approach. This provides a reference point that is comparable to Earth-based norms while acknowledging that astronauts confront additional dangers. Ground personnel, on the other hand, remain well below dosage limits and are mostly exposed to low-linear energy transfer (LET) radiation, with biological effects posing far less outcome uncertainty than space radiation.

#### Approaches for Understanding Radiation Sensitivity and Mitigation

The implication for precision medicine in radiation protection of astronauts is that specific gene mutations, gene expression patterns or biomarkers should be studied to identify radiation-sensitive and radiation-resistant individuals before space missions are taking place. Therefore, identification of useful radiation biomarkers of sensitivity is currently being developed to potentially and hopefully stratify and tailor, at term, radiation exposure schemes for individual astronauts, to further help and guide the decision process of specific space missions, considering higher risk exposures during extravehicular activities.

The term “radiation sensitivity” refers to an organism’s susceptibility to the effects of IR. Inadequate repair and/or misrepair of radiation-damaged genetic material of the cells (DNA), for example, due to deficient repair processes, may be the cause of radiation sensitivity. Each person’s radiation sensitivity or radiation resistance is determined by his or her genetic composition. Biomarkers and bioassays for measuring individual radiation sensitivity in the moderate to high dose range may become accessible in the next years, allowing for the routine and reliable identification of individuals with higher radiation sensitivity. Although these approaches for precision radiation protection in astronauts have the potential to refine the radiation exposure program of individual astronauts for future missions, prospective testing and validation in well-designed sets-up will be required before they are broadly implemented in the future. Gene expression through transcriptomics can also be utilized to define a radiation sensitivity index of astronauts that has the potential to be used to personalize mission programs and exposure to radiation. This would allow radiation protection to move toward a future of precision medicine based in part on genomic features of the individual, as it is important to recognize the challenge of human body heterogeneity which is composed of multiple cell types, each potentially harboring different radiation sensitivity (Rosen et al., 2000).

Space exploration is one of the most difficult and risky efforts to undertake; thus, decreasing the dangers of planetary EVAs is critical in allowing such tasks to take place. It is critical to define and categorize the risks in order to reduce them (Vuolo et al., 2017).

To ensure astronaut safety and mission success, it is imperative to also identify and mitigate the inherent risks and challenges associated with EVAs and to develop an individualized space suit according to these challenges. Prevention of hypercapnia prevention (increase of carbon dioxide partial pressure in the arterial blood >45 mmHg), thermal regulation and humidity control, nutrition, hydration, waste management, health and fitness, decompression sickness, radiation shielding, and dust mitigation are all factors in spacesuit design.

Although EVA performance has improved significantly, further research and development is still required to enable safer and more effective surface exploration activities in the future. In particular, as we continue to explore beyond low Earth orbit (LEO) and embark on missions back to the Moon and onward to Mars, it becomes critical to reassess EVA IR risks in the context of a planetary surface, rather than in microgravity. Spacesuits provide protection from UV rays, but they provide a very limited protection from all forms of IR. Therefore, research is required to investigate enhanced spacesuit materials with better IR protection characteristics. As IR sensitivity varies depending on biological sex, sizes, organs etc., a spacesuit should be designed on an individualized basis by modelling at the personal level (Thomas et al., 2012)*.*

### The Immune Response and Space Flights

#### Space Immunology: An Overview

The immune system plays a central role in our bodies’ surveillance of both outer and inner danger signals. Maintenance of immunity and the response to environmental stressors such as radiation involves a complex interplay between many physiological aspects including nutrition, endocrine regulation, bone marrow activity, exercise or sleep quality (Crucian et al., 2018). Dysregulation of the immune system during space flight has been detected by reactivation and shedding of latent herpesviruses in astronauts on the ISS (Mehta et al., 2017). Proposed mechanisms for immune dysregulation include reduced function and altered distribution of leukocytes and T helper type 2 (Th2) shifting of cytokine profiles (Mehta et al., 2013).

Interestingly, one study in particular has shown a transient reduction of thymopoiesis in 16 astronauts returning from flight, which would connect to changes in the T-cell response and the reduced ability to combat infections (Benjamin et al., 2016). On the contrary, another group was able to show that from a 6-month mission to the ISS, the B-cell counts and phenotypes were maintained in 23 astronauts suggesting that vaccination could be used as a preventive measure by using the “stability” of B-cell response in different treatment strategies (Spielmann et al., 2019).

Additionally, recent data of crew members revealed that the IgM antibody repertoire experienced significant changes during a 6-month mission on the ISS. Such modifications were quantified to be persistent even 4 weeks after landing and likely affected the specificities of IgM binding sites. These effects were individually different, correlated with changes in the V(D)J recombination process, responsible for creating antibodies, and coincided with a higher stress response that in some cases correlated with mission characteristics (Buchheim et al., 2020).

This interaction between stress and altered immune response as a function of long-duration exposure to space flight conditions was quantified by measuring a stress dependent release of endocannabinoids which showed an aberrant immune activation pattern seen in the elderly (“inflammaging”) (Buchheim et al., 2019). Therefore, there is a possibility that this type of exposure triggers an immune response that is highly individual, which may depend on experience and mission type and can result in long lasting changes.

After examining the medical records of crew members for 46 long duration missions on the ISS (20.57 crew flight years), Crucian et al. reported on the incidence of immune-related health adverse events (Crucian et al., 2016a). A striking 83% of crew members experienced a medical event with 46% of crew members reporting a “notable” event. Notable events were defined as prolonged duration, repeated or recurring and/or unresponsive to treatment. The connection between skin and the immune system made skin rashes the most commonly reported notable event (40%) followed by infectious diseases (29%) and atypical allergies (17%). The most prescribed medications during flight missions were antihistamines used for chronic conditions which persisted longer than 7 days (Wotring 2012). Characterization of on-orbit rashes manifested as redness with irritation and could be found in a variety of body locations. Whereas these skin rashes and allergic type of events seem to occur at the onset of missions infectious events tend to develop as the mission progresses from month three onwards. Interestingly, susceptibility to immunological events differed between astronauts with 11 crew members experiencing a single notable event, ten crew members experiencing two notable events, two crew members reporting three notable events, one crew member experiencing four notable events and one member even experiencing nine notable in flight events. Therefore, the reasons for interindividual variability in susceptibility to immune events during space flight requires further investigation through appropriate longitudinal non-invasive monitoring tools for prevention and early treatment.

#### Approaches for Understanding the Immunological Impact

##### Terrestrial Analogue Studies to Investigate and Predict Effects of Space Flight on Immune Response

Terrestrial analogue environments that mimic the extreme conditions of space flight such as bed rest studies, field deployment to Antarctica over winter and microgravity cell culture techniques have all been used to replicate the extreme environment of space flight (Crucian et al., 2014).

However, due to the complex interplay between the immune system and all other physiological systems as well as between the innate and the adaptive immune system, the investigation of single space related stressor (e.g., confinement) in these associated environments may be insufficient to replicate the exact conditions of space flight. Nevertheless, it offers the unique opportunity to establish standardized and controlled conditions (e.g., standardized nutrition protocols) to test for immune functional and molecular effects, or to study the effect of change of variables e.g., in cross-over study settings (Strewe et al., 2018). In a series of isolation studies in the past, crews were confined on Earth for either 105, 110 or 240 days and up to 520 days, the latter mimicking a full journey to Mars (MARS500-project) and the gradual effects of immune adaptation and overshooting reactivation pattern were observed (Kerrigan et al., 1989; Chouker et al., 2002; Yi et al., 2014)**.** It was observed that after 520 days of isolation the induced set of environmental pollutants and allergens enhanced cytokine responses in a setting of *ex vivo* antigen exposition (Yi et al., 2015) indicating a sensitization to allergens due to confinement. This is of great importance in the understanding of how a systemic response might be accumulating with time.

Other mission-relevant scenarios of real exposure to isolation conditions in the field have delivered data that indicate effects on the immune system that can be reproduced under such conditions where the effects of harsh environmental conditions in space exploration can be mimicked to some extent. For instance, the reduction of the atmospheric pressure resulting in hypobaric hypoxia, is one of the living conditions in future Lunar or Martian habitats. Though hypoxia has immune suppressive effects after a shorter duration of exposition, a month-long exposure leads to a state of higher immunological susceptibility to activation not yet observed in humans before in those environments. To which degree these type V immune allergic patterns are of relevance for disease needs to be further investigated (Feuerecker et al., 2014; Feuerecker et al., 2019).

Moreover, field isolation studies in the Antarctic and crew compositions of different sex help in identifying differences in stress and immune reactions proposing a sex relevant individualized risk management (Strewe et al., 2019). The investigations in several winter-over crews at the Neumayer III station in the Antarctic revealed that the stress hormone cortisol during winter showed significantly higher concentrations in females and was independent of differentially enhanced psychological stress levels as quantified by questionnaires in the mixed crews, respectively. Interestingly though, other endogenous stress mediators, such as endocannabinoids and N-acylethanolamides, increased significantly in both sexes and were consistently elevated during the confinement as well as the cytokine profiles after *in vitro* stimulation. This was also true for significantly elevated lymphocyte counts during confinement, indicating a sex independent immune status change in these cohorts (Strewe et al., 2019).

##### Biomarkers as a Tool for Detecting Immunological Changes in Space

Biomarkers for immunocompromised states can be very helpful if they are strong in their diagnostic value, easily accessible and can be collected repeatedly. This is of special value for monitoring space crew and for addressing the impact of stressful conditions in the course of a mission, adaptation to new environments and, of increasing importance, for monitoring the effects of countermeasures. Here, quantification of different and otherwise dormant (e.g., herpes simplex virus (HSV)) in saliva and other biospecimens have shed light into antiviral immune changes in space crew (Mehta et al., 2014; Mehta et al., 2017; Rooney et al., 2019). These biomarkers have a dual role, as salivary viruses are also of pathogenic nature and potentially infectious (e.g., varicella zoster virus (VZV)) (Cohrs et al., 2008). Moreover, the continuous monitoring in several subsequent investigations on the ISS revealed, that these surrogate markers of immune dysfunction play a significant role in detecting improvements in exercise regimen of astronauts. Advances in the exercise regimen has been associated with fewer cases of viral shedding in United States operational segment crews in the past few years (Agha et al., 2020b; Crucian et al., 2020).

The signaling pathways and gene regulators responsible for initiating a transition from latency to reactivation of dormant viruses remain to be identified. While singular switch regulating genes may have been identified, such as BamHI Z fragment leftward open reading frame 1 (*BZLF1*) acting as the immediate early gene in Epstein Barr Virus infected B lymphocyte culture (Wen et al., 2007), their upstream/downstream signaling pathways or regulatory functions during lytic or latency phase are less known. Recent publications hint at a possible epigenetic regulation of transcription during the latency phase in VZV and HSV through so called latency transcripts and reactivation is associated with the modification of bound histones (Kennedy et al., 2021). To better understand the genetic pathways and regulators responsible for reactivation of dormant viruses, a transcriptome or epitome analysis on inflight blood samples before and during shedding, could be utilized to identify potential early markers before the virus is present in saliva. A whole transcriptome analysis using RNAseq on blood samples of the winter-over crew at the Antarctic Concordia research station revealed a strong susceptibility to viral infections at the RNA level due to strong suppression of the interferon pathway (Buchheim et al., 2020). However, previously published protein levels of interferon gamma (IFNy) were unaltered after spaceflight (Crucian et al., 2014). We would therefore recommend performing individual genetic analyses in astronauts in the future as analogue studies may not translate to spaceflight.

Recently, AMPs were quantified along a 6-month mission to the ISS reporting that long-duration space flight alters the concentration of AMPs in saliva linking this to altered corticoid levels. Importantly, and predicted by prior investigations, they showed that there were differences in the astronauts AMP alteration depending on whether the astronaut was new or an experienced and could also linked the reactivation of latent virus to EVAs known to trigger a strong stress response (Agha et al., 2020a). This implies that repetitive exposure to extreme environmental conditions in space are of importance and add to the evidence in favor of finding new analytical formats for immunological response based on the individual. Interestingly, no differences were found between the group of virus shedders and non-shedders indicating independent immune regulatory processes (Agha et al., 2020a).

Nutrition and diet can influence immune functions especially regarding oxidative stress. To counteract oxidative stress and to reduce the harmful effects of IR in astronauts, it is currently recommended to supplement scavenger vitamins, trace elements and minerals in the dietary intake of astronauts (Smith and Zwart 2008). Dietary and antioxidant defenses play a protective role in muscle cells by reducing associated oxidative damage to lipids, nucleic acids, and proteins (Bergouignan et al., 2016; Catalano 2016). Strategies to counteract the detrimental effects of excess free radicals by supplementing an antioxidative cocktail in manned missions have been unsuccessful (Gomez et al., 2021). The authors conclude that this is due to “the absence of prior genetic testing that determines each astronaut’s capacity to produce endogenous antioxidants”, emphasizing the importance of an individualized approach to dietary recommendations.

There is a large body of evidence supporting vitamin D supplementation for the prevention of bone density loss. Increasing the daily dosage of vitamin D from 400 to 800 units in conjunction with exercise is the only evidence-based countermeasure able to maintain bone mass (Smith et al., 2012). However, there are no studies that have investigated vitamin D and astronaut immune function.

New evidence has emerged linking the gut microbiome and regulation of bone physiology in health and disease potentially bridging the gap between bone density loss and immune alterations. It has recently been demonstrated that activation of inflammation and innate immunity by gut microbiota components increases the production of TNFα and the osteoclastogenic factor RANKL (receptor activator of nuclear factor kappa-B ligand) in bone leading to reduced cortical thickness (Ohlsson et al., 2017; Ibanez et al., 2019). Prebiotics, which improve the gut microbiome, have been shown to improve calcium uptake and bone structure in humans (Abrams et al., 2005) and potentially have a role in protection against bone loss (Pacifici 2018). Interestingly, prebiotics have also been shown to regulate peripheral Treg population in mice (Smith et al., 2013). Treg levels are reduced in astronauts after long duration missions, which may account for hypersensitivity observed towards recall antigens after return (Buchheim et al., 2019). Further research should be undertaken to investigate the effects of nutritional changes and immune function in astronauts.

With regards to *in vitro* modelling techniques, the organ-on-a-chip approach has been presented as an option for modelling organs of the immune system (e.g., skin, gut, spleen, thymus) and their associated immune cellular components (Shanti et al., 2018). Due to the small size, this technique could be a way to test for immunotoxicology and immune response in general in space flights and even become part of countermeasures.

Another way of examining the effects of space flights would be to detect and use biomarkers. Here Paul et al. suggested that the neutrophil-leukocyte ratio could be used as a biomarker for immune status in astronauts (Paul et al., 2020). Other biomarker candidates can be viewed in Table 1, where we have included those reviewed and summarized above. As the immune response is multifaceted it would also be of interest to use bioinformatic tools to advance the understanding and connection of the immunological responses on Earth in analogs such as those explained here with those in space.

**TABLE 1:** Established and potential biomarkers in astronauts.

Target	Effects and functions	Sample type	References
Viral DNA	Indirect monitoring for low immunity	Saliva	Mehta et al., 2014, Mehta et al., 2017, Rooney et al., 2019
Cortisol	Major stress hormone, correlated with EVAs, circadian rhythms	Saliva	Agha et al. (2020a)
(s)IgA	Immunoglobulin; preformed defensin on mucus membranes	Saliva	Agha et al. (2020a)
Lysozyme	Antibacterial; inactivates viruses	Saliva	Agha et al. (2020a)
LL-37	Antibacterial; immune modulating activity; cationic properties	Saliva	Agha et al. (2020a)
*BZLF1*	Immediate early gene during EBV infection	Cell culture	Wen et al. (2007)
Endogenous antioxidants	Individual capacity to generate endogenous antioxidants	Blood	Gomez et al. (2021)
Exercise	Preventive measure to avoid viral shedding	Diary, questionnaire	Agha et al. (2020b)
Neutrophil-leukocyte ratio	Direct monitoring of immune function	Blood	Paul et al. (2020)

Abbreviations: BZLF1, BamHI Z fragment leftward open reading frame 1; EBV, epstein barr virus; EVAs, extravehicular activity; (s)IgA, soluble immunoglobulin A.

##### Mitigation Strategies: Immune Directed Countermeasures

Both pharmacological and non-pharmacological immune-directed countermeasures have been proposed by an international panel of experts (Crucian et al., 2018). These include pre-mission screening for immune function, clinical history, herpes virus serology and vaccination. During the mission astronauts should be issued personalized recommendations for diet and nutritional supplementation (e.g., glutathione) as well as exercise regimes to maintain the immune system and reduce stressors. Radiation countermeasures will also need to be incorporated, as discussed earlier, to protect the immune system during space flight. The medicine formulary available to astronauts to treat immune system dysregulation (e.g., antihistamines, antivirals) should also be personalized and incorporate pharmacogenetics (discussed in next section) where appropriate, to maximize efficacy and minimize potential adverse drug reactions (ADRs). If possible, regular or live monitoring of immune parameters, such as viral load and white cell count, may enable pre-emptive recognition of impending immune dysfunction so that active countermeasures can be deployed before illness develops.

The full implementation of the measures described above will require advances in technology and development of clinical decision support systems (CDSS) that can integrate the preflight information in real time with data received from astronauts during space flight.

## The Use of Medicines in Space

### Overview

Human space flight takes place in remote and physiologically challenging conditions with medical provision constrained by the expertise of the crew and interventions, such as medicines, limited by mass and volume restrictions. Prevention, through screening and countermeasures, represents one of the most successful measures to mitigate physiological impairment and ill health. Despite these prevention measures, astronauts can still experience ill health or injury. The risk of a serious medical emergency during space flight has been estimated at 0.06 per person-year of flight or one event per 68 months (Houtchens 1993). Additionally, astronauts also experience minor symptoms including space motion sickness, skin rash and insomnia requiring pharmacotherapy (Hodkinson et al., 2017). The frequency of medication administration during human space flight is not comprehensively monitored but estimates suggest an average of 23.1 medications per crew member over the course of a mission on the ISS. The most commonly administered medications were analgesics, decongestants and sleep aids (Blue et al., 2019). It is therefore important that medications which form part of the medical package are effective and the incidence of adverse effects minimized.

### Drug Pharmacokinetics in Space

Exposure to the space environment leads to physiological adaptations that can affect the pharmacokinetics of medicines. Changes to the gastrointestinal tract can alter the bioavailability of medicines through alteration of gastric emptying rate, intestinal transit time and absorption (Davis et al., 1993). Microgravity leads to loss of physiological gradients of the arterial, venous and microcirculatory pressure resulting in fluid shifts from lower to upper part of the body and decrease in blood volume into tissues (Charles and Lathers 1991). Taken together with alterations in plasma proteins and endothelial cell function, the distribution of medicines in space travel is likely to be altered but there is limited evidence from space flight studies (Leonard et al., 1983; Maier et al., 2015). The liver is the major organ responsible for metabolism of xenobiotics and drugs. There is evidence to suggest increased hepatic blood flow during space flight with increase in liver size (Grigoriev et al., 1991). However, the exact relationship between liver alterations and metabolism of medications in space flight has not been fully investigated. Many drugs are eliminated by the kidneys. Weightlessness in space has been shown to attenuate urine output following an oral water load compared with head down bed rest studies (Norsk et al., 2000). It is difficult to predict how these changes will affect the PK of individual medicines during space flight.

#### Examples of Pharmacokinetic Studies During Space Flight

Very few pharmacokinetic studies have been conducted during space flight and all have relied on PK measurements using saliva samples (Cintron et al., 1981; Kovachevich et al., 2009). During human space flight, acetaminophen (2 × 325 mg) tended to show decreased Cmax on flight day (FD) 0 and an increased Cmax on FD2 and FD3 whilst Tmax tended to increase although there was significant variability within the data (Putcha and Cintron 1991). Intersubject variability was minimal preflight suggesting that PK responses to space flight show variability between individuals.

More recently, a study compared single dose 500 mg acetaminophen tablets and encapsulated forms in ten astronauts, divided into two groups of five men, preflight and during long-term space flight (Kovachevich et al., 2009). Salivary concentrations were determined up to 6 h. The tablet form demonstrated reduced rate of absorption but substantial increase in bioavailability during space flight compared with terrestrial conditions. The encapsulated form demonstrated decreased time of absorption whilst the elimination half-life, retention time and distribution volume increased considerably but overall bioavailability was similar in comparison with terrestrial conditions. The elimination curves were similar particularly for ground studies.

Scopolamine in conjunction with dextroamphetamine is a commonly used medication to counteract space motion sickness (Davis et al., 1993). A PK study in three astronauts taking scopolamine 0.4 mg and dextroamphetamine 5 mg demonstrated significant inter-astronaut variability (Cintron N et al., 1981). In one astronaut a reduction in Cmax was observed with prolonged Tmax; the second astronaut showed increased Cmax with unchanged Tmax whilst in the third crew member there was a decrease in Cmax and increase in Tmax at FD0-1 and increased Cmax and decreased Tmax on FD 2–3. In this astronaut, the PK profile for scopolamine appeared to be abnormal at FD0-1, with two concentration peaks observed, suggesting that mission day may also have an impact on PK studies. There were significant gaps in PK sampling during Space flight due to dry mouth and as a side effect of the scopolamine.

Taken together, these data suggest that the rate of exposure to medicines is more likely to be affected by microgravity rather than the amount of exposure. However, these studies were of short duration and cannot be extrapolated to longer flight missions. Salivary PK samples may not be as accurate as blood PK samples. However, blood sampling would be constrained by the technical challenges of collection, storage and return of samples during human space flight. Additional pre-clinical organ or tissue-based methods should be developed and used to supplement the clinical endpoints.

### Approaches and Countermeasures in Space Pharmacology

#### Pharmacogenetics as Part of the Personalized Medicine Approach

Pharmacogenetics is the study of genetic variability and its influence on drug response. Variation in drug metabolizing enzymes, transporters, receptors and ion channels can alter the efficacy and risk of adverse reaction to a medication. The Food and Drug Administration (FDA) publishes a list of pharmacogenetic associations with gene-drug interactions and therapeutic recommendations where these exist (US-Food-Drug-Administration 2021). Eight medicines present on the FDA table of pharmacogenetic associations also form part of the formulary on the ISS (Table 2). The majority of these are associations with polymorphisms in cytochrome P450 genes. *CYP2D6* is a very important pharmacogene that is highly polymorphic and responsible for metabolism of up to 25% of medicines, including many psychotropic drugs which are also used for the treatment of motion-sickness (for reference of the CYP2D6 psychotropic substrates and pharmacogenetic variability, see Stingl Mol Psychiatry 2013) (Owen et al., 2009; Stingl et al., 2013). The ability of the CYP2D6 enzyme to metabolize substrates is dependent on the haplotype and subjects are classified into four categories depending on enzyme activity: ultra-rapid metabolizers (UM), normal (extensive) metabolizers (EM), intermediate metabolizers and poor metabolizers (PM) (Taylor et al., 2020). Population frequencies for different CYP2D6 alleles demonstrate significant variability across World populations. For example, PM status was observed in 8.45% of Europeans, 5.38% African Americans and only 0.84% of East Asians (Gaedigk et al., 2017). Astronauts are derived from diverse backgrounds and pharmacogenetic screening will need to include rare variants to ensure equality and equitable access.

**TABLE 2:** Drugs that are part of the ISS formulary with FDA acknowledged pharmacogenetic associations.

Drug	Indications	Gene	Affected subgroup	FDA description of gene-drug interaction
Aripiprazole	Psychosis	*CYP2D6*	Poor metabolizers	Higher systemic concentrations and higher adverse risk. Dosage adjustment is recommended
Diazepam	Seizure, sleep disturbance	*CYP2C19*	Poor metabolizers	May affect systemic concentrations
Meclizine	Motion sickness	*CYP2D6*	Ultrarapid, intermediate, or poor metabolizers	May affect systemic concentrations. Monitor for adverse reactions and clinical effect
Metoprolol	Heart failure, hypertension	*CYP2D6*	Poor metabolizers	Results in higher systemic concentrations
Omeprazole	Reflux	*CYP2C19*	Intermediate or poor metabolizers	Results in higher systemic concentrations
Sulfamethoxazole and trimethoprim	Infection	*NAT (nonspecific)*	Poor metabolizers	May result in higher adverse reaction risk

CYP, cytochrome P450; NAT, N-acetyltransferase.

Many drugs used in psychiatric illness, such as antidepressants, antipsychotics, and mood stabilizers, are affected by pharmacogenetic polymorphisms, with CYP2D6 being involved in the metabolism of approximately half of the commonly prescribed psychotropic drugs (Mulder et al., 2007). Since differences in plasma concentration, due to variability in drug clearance, often vary by ten-fold or more, pharmacogenetic dose adjustments have been issued (Swen et al., 2011; Hicks et al., 2013; Stingl et al., 2013). Inadequate drug exposure causes a risk for nonresponse or toxicity depending on the therapeutic range of the drug. In antidepressant drug treatment, PM for CYP2D6 have been associated with longer time requirements to find the most appropriate drug and with more frequent drug switches (Bijl et al., 2008). PM may suffer more frequently from ADRs than EM require longer hospital stays, whereas ultrarapid metabolizers (UM) have a higher risk of therapeutic failure (Chou et al., 2000; Bijl et al., 2008; Ruano et al., 2013). Pharmacogenetic guidelines are available with dosing recommendations for specific drug-genotype pairs (Relling and Klein 2011; Hicks et al., 2013). Of all genes involved in these evaluations, *CYP2C19* and *CYP2D6* have been shown to mainly affect the outcome and the risk of ADRs of antidepressants and antipsychotics. The effect of the CYP2D6 genotype on adverse drug effects and nonresponse, as well as on non-adherence during treatment with CYP2D6-dependent antidepressants, has been shown in several studies (Jukic et al., 2018; Jukic et al., 2019; Braten et al., 2020; Milosavljevic et al., 2021).

The FDA pharmacogenetics report is not exhaustive and there are many other pharmacogenetic associations reported in the literature with medicines that form part of the ISS formulary. For example, sleeping aids, such as zolpidem, zaleplon and diazepam, were the most commonly used medicines by US astronauts on the ISS (Wotring 2015). A study in Han Chinese patients has shown that CYP3A4*18 (increased CYP3A4 activity) and CYP2C19*2 (reduced CYP2C19 activity) significantly affect the metabolism of zolpidem with the potential to reduce efficacy and increase toxicity (Shen et al., 2013). Astronauts that possess these polymorphisms may need their dosage adjusted or treatment with an alternative therapeutic agent. The major histocompatibility complex encodes human leukocyte antigens (HLA) which play a key role in the regulation of the adaptive immune response through presentation of processed peptide antigens (Wieczorek et al., 2017). Carriage of specific HLA alleles has been associated with susceptibility to severe adverse effects to certain medicines. Trimethoprim-sulfamethoxazole (TMP-SMX) is an antibiotic available in the ISS formulary. Carriage of HLA-B*14:01 has been associated with TMP-SMX drug induced liver injury in European Americans and HLA-B*35:01 may be a risk factor for African Americans (Li et al., 2021). Similarly, HLA-B*15:02, HLA-C*06:02 and HLA-C*08:01 have been associated with TMP-SMX-induced severe cutaneous reactions (Kongpan et al., 2015). These types of reactions can be life threatening and would be catastrophic if they occurred during space flight. Prospective astronauts could be HLA genotyped prior to the mission and a personalized formulary created to avoid HLA-associated risk medicines.

**FIGURE 5:**
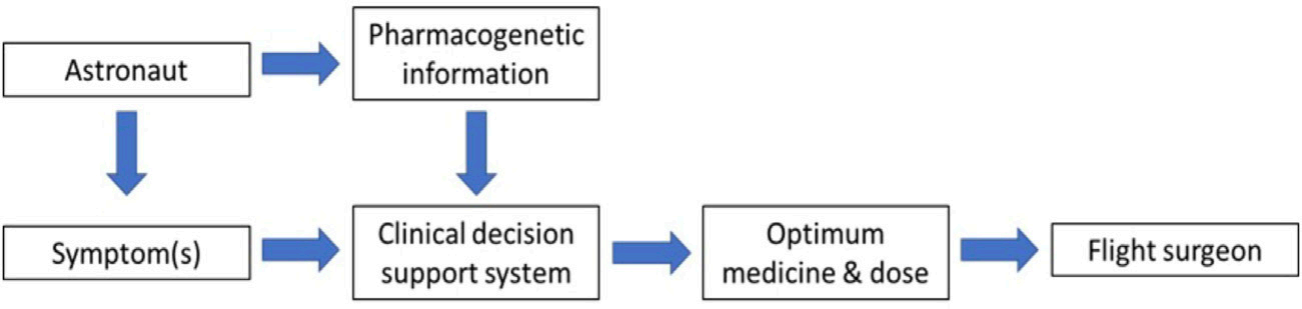
Incorporating astronaut pharmacogenetic information into clinical decision support to help the flight surgeon.

The flight surgeon is responsible for ensuring the health, safety and performance of astronauts from selection, training and space flight through to postflight rehabilitation and long-term health (Houtchens 1993). Pre-flight pharmacogenetic screening from astronauts could be incorporated into electronic health records as part of CDSS designed to support the flight surgeons in selecting the most appropriate medicines and dosages for each astronaut to maximize efficacy and reduce toxicity (Figure 5). One of the challenges for pharmacogenetics is translating pharmacogenetic information into actionable prescribing decisions. Not all of the FDA pharmacogenetics associations are accompanied by prescribing recommendations. Organizations such as the Clinical Pharmacogenetics Implementation Consortium (CPIC) produce clinical guidelines and prescribing recommendations for pharmacogenetic associations. Space agencies will need to decide on the strength of such evidence and whether or not to incorporate it into their CDSS. The interface for CDSS will be critical and lessons should be learned from terrestrial implementation of similar systems such as the eMERGE network (Herr et al., 2019). Flight surgeons and astronauts will be critical to this process. The CDSS will need to be regularly reviewed and updated to incorporate the latest pharmacogenetic research.

#### Countermeasures

It is surprising that so few PK studies have been conducted during space flight given the fundamental relationship between PK and the therapeutic and toxic effects of medicines in such a high stakes environment. We suggest that PK studies with blood sampling and pharmacogenetic screening are prioritized for future LEO missions (e.g., ISS). Space agencies should focus on medicines that are most frequently used (e.g., sleeping agents) and which have the most potential for harm if they were to be ineffective (e.g., antibiotics). Interestingly and of importance for PK monitoring are the use of new micro and nanotechnologies such as microneedle biosensors that allow real time minimally invasive monitoring of drug levels with the potential for personalized dosage adjustments (Rawson et al., 2019). The data derived from these missions could be incorporated into physiologically based PK models allowing extrapolation of microgravity PK effects to other medicines (Srinivasan et al., 1994). Additionally, this form of minimal invasive procedure would pave the way to use this technique to deliver drugs transdermally, with motion sickness treatment being one of the most obvious candidates to start with. Here the incorporation of skin equivalents in preclinical PK (and PD) trials would be beneficial. Other formats for the delivery of drugs could include the use of nanoparticles which can more effectively deliver the drug or substance to the tissue or organ needed or remodel microenvironments in tissues or organs in a non-invasive way (Mitchell et al., 2021). This technique is already revolutionizing the way drugs are developed and should certainly be considered for space flight missions.

Integration of pharmacogenetics and CDSS allied with better understanding of PK for medicines administered in microgravity will be critical to ensure that the right medicines are administered at the optimum dose to the astronaut to treat and maintain health.

## Discussion and Vision for the Future

The aim of personalized medicine is to provide health care that takes into account an individual’s unique physical, genetic, clinical and sociodemographic characteristics in order to predict disease and treatment response on an individual level. Traditionally, evidence-based medicine is practiced based upon population level data and how an “average” individual may behave, but information from this approach is not readily applicable to an individual patient. With the advent of new technologies, electronic healthcare systems, favorable economic policies, researcher/clinician education and public engagement it is envisioned that personalized medicine may become reality in the next 10 years (Vicente et al., 2020).

Space medicine and the healthcare of astronauts is an area where personalized medicine will become increasingly important especially in the context of longer exploration class missions to Mars. We have explored some of the risks experienced by astronauts with respect to skin barrier impairment, the dangers of IR, immunological dysfunction and response to medicines. We have demonstrated *in vitro* and *in vivo* evidence to suggest that adaptation to microgravity and susceptibility to the risks of the space environment demonstrate significant inter-individual variability. This variability provides the motivation to develop individualized countermeasures. We have also made recommendations with regards to future research.

Measurement of biomarkers has been used as a tool in personalized medicine to predict the outcome of treatments (Zenner 2017). MicroRNAs represent one such biomarker concept and have emerged as a new tool in disease research due to its cell specificity and stability in blood, urine and saliva (Turchinovich et al., 2012). However, the concept of a molecular biomarker does not represent a “true” individual analytical approach as no connections to and between an individual’s epigenetics, protein and metabolic profile or the environment has been considered. Variation in biomarker levels have been shown to be significantly influenced by genetics and lifestyle. Incorporating personalized cut offs taking into account these factors could increase the sensitivity for prediction of clinical endpoints (Enroth et al., 2014).

**FIGURE 6:**
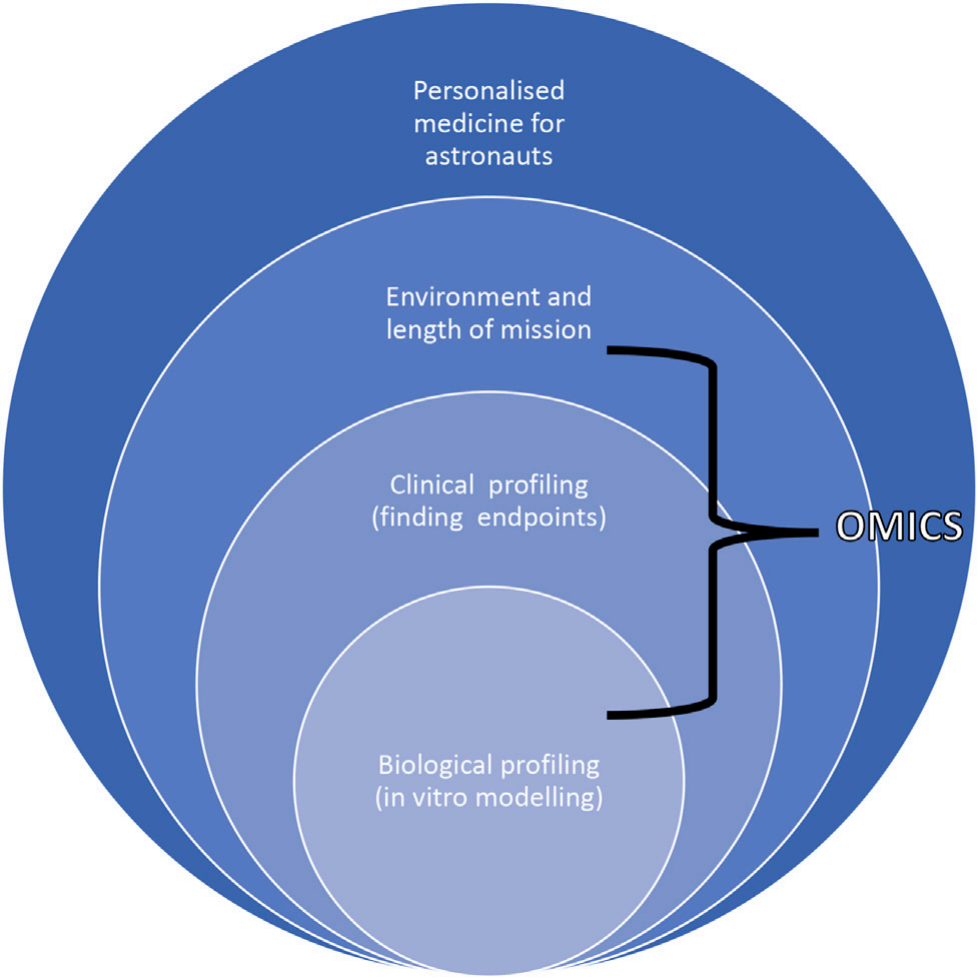
Scheme describing the dependence of biological and clinical profiling with environmental cues and length of mission to accomplish personalized approaches for astronauts highlighting how omics tools can be used to move from correlations to causation.

Astronauts would be an optimal group for this “new” molecular biomarker mapping approach as it is possible to obtain rich clinical data during the pre-mission phase to develop a detailed individual biomarker network. These biomarkers can be monitored during and after missions and correlated with clinical endpoints to preemptively initiate countermeasures where negative effects are predicted. Environmental factors such as mission duration and tasks such as EVA would also be included. This new molecular analysis format would open the way to an innovative, although complex, tool to be used in clinical settings here on Earth as well (Figure 6).

With the advent of omics and high throughput technologies it is possible to gather large amounts of data from single individuals. The NASA twins study measured physiological, telomeric, transcriptomic, epigenetic, proteomic, metabolomic, immune, microbiomic, cardiovascular, vision related and cognitive data in a pair of monozygotic twins enabling comparison of the impact of prolonged space flight environment (340 days) on one twin to the simultaneous effects of the terrestrial environment in a genetically matched individual (Garrett-Bakelman et al., 2019). There were extensive multisystem changes during human space flight, including on return to Earth, but the majority returned to a preflight state during the study. However, some gene expression levels, chromosomal inversions, increased short telomeres and attenuated cognitive function persisted beyond the study. This study provides mechanistic insight into changes that occur during space flight and the potential to provide more effective countermeasures. Integration of omics data is concluded to be critical for translation of personalized medicine into clinics, supporting our suggestions.

Collating, storing and integrating data from multiple sources and experiments is a significant challenge requiring bioinformatics and biomathematics expertise. The NASA Gene Lab Project provides a platform to collect, collate and provide access to genomic, transcriptomic, proteomic and metabolomic data from biological specimens flown in space or exposed to simulated space stressors (Berrios et al., 2021). The International Standards for Space Omics Processing (ISSOP) consortium has recently been set up to develop, share and encourage sample processing standardization and metadata normalization of space flight omics experiments (Rutter et al., 2020), providing an encouraging first step into the challenges ahead.

Due to the complexity of these types of approach, it will require the development of CDSS, remote assessment tools and machine learning technologies to allow integration of the data and real time interaction between flight surgeons and crew. Omics-based biomarkers for environmental health studies have already been explored and the lessons learned are also applicable to space medicine (Espín-Pérez et al., 2014). For this approach to be successful, the hypothesis and targeted-analyses driven format used until now in space medicine would need to be redesigned to an omics/clinical endpoint format which detects off-target or unanticipated effects, as those might be of importance while exploring new extreme environments. The successful implementation of these systems in human space flight and their combination can then be repurposed to medical care in extreme terrestrial environments (e.g., submarine missions) and other areas with limited resources.

Such an approach could be used to determine radiation protection thresholds at an individual level. At present radiation protection thresholds are determined without completely incorporating the tremendous advances in radio-biology into individual radiation susceptibility measures. Instead, the IR threshold is typically determined regardless of the molecular features of the individual (worker or astronaut). In view of the future missions to the Moon and to Mars, it is imperative to discuss the opportunity to widen the window of radiation protection in the era of precision medicine by combining technology-driven radiation precision with biology-driven approaches and how advances in biomarker research and “Big Data” can be leveraged to tailor radiation protection issues to each individual astronaut in an effort to decrease IR impact. While these and other approaches to precision medicine should be tested to improve radiation protection of astronauts, the limits to personalized medicine that arise from variations between individuals and missions (duration, radiation dose and dose rates, exposure, solar activity) must be appreciated.

It should also be noted that it is important to explore different horizons to protect astronauts from cosmic radiation and improve human radiation resistance, beside the spacesuit, which can be done using recent developments in biotechnology. These include the possibility of making genetic modifications to humans through the use of advanced gene editing techniques along with current knowledge of molecular pathways that address the DNA damage caused by IR, as well as other possible treatments, such as regenerative medicine, low-dose radiation adaptation, the use of organic compounds, hypostasis (considerable slowdown of all the vital processes in the body) or a combination thereof (Cortese et al., 2018). Nevertheless, these ideas present novel ethical issues and ensuing debates are emerging especially considering opportunities to enhance astronauts’ performance by genetic manipulation either employed as countermeasures (i.e., to withstand higher radiation burden) or to augment individuals’ resilience in a harsh environment (Gibson 2006; Milligan and Inaba 2020; Munevar 2020).

By using personalized *in vitro and ex vivo* modelling, representing an organ or tissue from each astronaut could be used to investigate pharmacological, tissue repair and environmental changes (e.g., microgravity, radiation), allowing the study of an individual’s response in a preclinical setting, without compromising the direct well-being of the astronaut. These astronaut specific organ models could be exposed to extreme environmental conditions pre-mission enabling flight surgeons and astronauts to be aware of individual risks and responses before embarking on space missions. These organ-on-a-chip models are already being used to measure terrestrial environmental toxicity and could be repurposed for space flight missions and used to test the effectiveness of proposed countermeasures without using up excessive capacity during flight missions (Yang et al., 2021).

With regards to tissue repair other modeling techniques might need to be explored. The function and use of 3D-bioprinting of tissues for repair of anatomical injuries will need to be taken into account. Another aspect of this is the longevity of the models and repair tissues, which is extremely connected to their biological stability. To support long lasting regeneration and biological accuracy, the models and tissue/organs used for repair would need to also be composed of biological material that does not promote degradation or artificial aging of the tissue. Human space flight is classified as travel greater than 100 km above sea level and is divided into three categories: 1) suborbital, 2) LEO (e.g., ISS), and 3) exploration class missions (e.g. missions to the Moon and Mars) (Hodkinson et al., 2017). These all represent distinct time and level of exposure to extreme conditions. The level of personalized medicine integration required will therefore be dependent on the type and duration of mission. Suborbital space flight, including space tourism, will require minimal personalized medicine compared with longer term missions which will be associated with greater risks including EVAs. Additionally, medical evacuation in cases of emergency, will require several days for missions to the Moon and months for missions to Mars emphasizing the importance of personalized medicine integration to maintain crew health and minimize adverse events.

In all these new ideas and endeavors, there are ethical issues that go beyond the human experimentation principles set out in the Declaration of Helsinki on ethics for medical research involving humans (World Medical Association, 2001; WHO, 2001) and these require thorough deliberation and oversight. There are concerns regarding pre-flight screening and whether there is effective “informed consent” of astronauts given the high number of unknowns in space flight (Hobe and Popova 2017; Koepsell 2017; Legato 2019). In a short-term perspective these issues have been managed within existing legal frameworks through the principles of the Declaration of Helsinki, National and International practices and negotiated within the Space Agencies. Current practices are already under strong scrutiny (Kahn et al., 2014) and in a longer-term perspective, ethical considerations have an important role in shaping the design and development of new methodologies of personalized medicine for space exploration (Gibson 2006; Langston 2018; Legato 2019).

In conclusion we have shown that responses to space flight demonstrate significant interindividual variability. Personalized medicine should be incorporated into space medicine to take into account these individual differences. With new technologies, such as individualized *in vitro* modelling, 3D-tissue printing and high throughput sequencing, it is possible to start gathering large amounts of data enabling personalized predictions of risk and individualized countermeasures. Platforms such as NASA’s Gene Lab and ISSOP provide recommendations for standardized data collection, processing and storage, which are fundamental to enabling a personalized approach. As these systems mature, bioinformatics and CDSS will integrate this information allowing real time interaction between flight surgeons and crews to maintain the health of the astronauts. These sophisticated biomedical tools, countermeasures and technologies have significant potential to benefit terrestrial health (e.g., telemedicine) which also include further understanding of environmental-physiological processes such as effects of IR on aging, immune dysregulation and skin fragility to name a few. Personalized space biomedical research can be a new gateway to understand the multidimensional reality that our bodies are exposed to.
